# Single-Cell Identification of Melanoma Biomarkers in Circulating Tumor Cells

**DOI:** 10.3390/cancers14194921

**Published:** 2022-10-08

**Authors:** Reilly Fankhauser, Matthew Chang, Zachary Garrison, Rachel Berryman, Olivia M. Lucero, Allison Fuiten, Nicholas DePatie, Hilary Seifert, Rajan P. Kulkarni

**Affiliations:** 1 Department of Dermatology, Oregon Health & Science University, Portland, OR 97239, USA; 2 Cancer Early Detection Advanced Research Center (CEDAR), Knight Cancer Institute, Oregon Health and Science University, Portland, OR 97239, USA; 3 Knight Cancer Institute, Oregon Health and Science University, Portland, OR 97239, USA; 4 Operative Care Division, U.S. Department of Veterans Affairs Portland Health Care System, Portland, OR 97239, USA

**Keywords:** melanoma, circulating tumor cells (CTCs), liquid biopsy, negative enrichment, single-cell RNA-seq, microarray, immune checkpoint blockade

## Abstract

**Simple Summary:**

Identification and investigation of cancer biomarkers is a pivotal area of modern cancer research. One such biomarker that has garnered considerable attention in the field is a class of free-floating tumor cells found in the blood known as circulating tumor cells (CTCs). Existing methods for CTC isolation tend to isolate cells based on a single marker enrichment that fails to capture the full heterogeneity that is commonplace in tumor cell populations. By incorporating a broader antibody cocktail, we designed a new approach to isolate CTCs for single-cell RNA analysis. Using common melanoma markers, a consistent and cost-effective approach was developed that we demonstrate to be successful with both patient and cultured cells. This methodology provides a framework to capture a more heterogeneous population of CTCs with wide applications in both research and clinical fields.

**Abstract:**

The current standard for investigating tumors is surgical biopsy, which is costly, invasive, and difficult to perform serially. As an adjunct, circulating tumor cells (CTCs)—cells that have broken away from the primary tumor or metastatic sites—can be obtained from a blood draw and offer the potential for obtaining serial genetic information and serving as biomarkers. Here, we detail the potential for melanoma CTCs to serve as biomarkers and discuss a clinically viable methodology for single-cell CTC isolation and analysis that overcomes previous limitations. We explore the use of melanoma CTC biomarkers by isolating and performing single-cell RNA sequencing on CTCs from melanoma patients. We then compared transcriptional profiles of single melanoma CTCs against A375 cells and peripheral blood mononuclear cells to identify unique genes differentially regulated in circulating melanoma tumor cells. The information that can be obtained via analysis of these CTCs has significant potential in disease tracking.

## 1. Introduction

Circulating tumor cells (CTCs) were first identified over 100 years ago [1]. Since their discovery, CTCs have been recognized as key drivers of metastasis and as useful biomarkers for studying disease status, progression, and evolution [2,3,4]. Profiling CTCs offers many advantages. In contrast to solid tumor biopsy, CTCs can be accessed through a minimally invasive blood draw, or “liquid biopsy.” CTCs may be released from both primary tumor and secondary metastases, including potentially micro-metastatic foci. Thus, CTCs can provide information about both intra- and intertumor heterogeneity [5]. Furthermore, some tumors are not surgically resectable, or are too small to be identified on a scan. In the absence of primary tumor material, CTCs may offer an alternative means to investigate tumor biology.

After the discovery of CTCs in 1869 [1], the field transitioned to refining effective methods for isolating these cells from liquid biopsies. Those pursuits led in part to the creation of the CellSearch System method [6], which has since prompted more clinical-based research around CTC utility as biomarkers of disease. Using this approach, Cristofanillli et al. demonstrated the predictive capabilities of CTC counts to forecast progression-free survival in metastatic breast cancer patients [7]. That study yielded a 5 CTC/7.5 mL threshold that correlated strongly with both progression-free as well as overall survival. Another study conducted by Nolé et al. similarly analyzed metastatic breast cancer patients throughout treatment and found a statistically significant link between those with greater than five CTCs and increased risk of disease progression [8].

However, the use of CTC counts as a biomarker has not shown survival benefit or helped to guide treatment options. The field has largely pivoted towards genetic analysis of CTCs to yield more specific predictive information and that may yield potentially actionable biomarkers that could be useful in the future to guide treatment decisions.

More applicable studies focused on melanoma have gone further to demonstrate genetic biomarkers that can be obtained via CTCs. Hoshimoto et al. conducted multimarker RT-quantitative PCR on CTCs obtained from over 300 melanoma patients and identified *MART-1*, *MAGE-A3*, and *GAlNAc-T* as predictive biomarker genes [9]. The potential of CTCs for clinical utility has been increasingly demonstrated by these and other similar studies.

As the potential clinical utility of CTCs has become more broadly recognized, much effort has been placed on developing tools to isolate and interrogate these cells of interest [10]. There are fundamental challenges that must be overcome in order to successfully isolate CTCs. Principally, CTCs are rare. When isolating them from whole human blood, CTCs are isolated in a ratio of one to every million or 10 million peripheral blood mononuclear cells (PBMCs) [11,12,13]. Previous methods for CTC enrichment fall into two broad categories: label dependent or label independent [14].

Label-independent methods rely on the size, density, or deformability of CTCs for enrichment. In many cancer types, the diameter of CTCs is generally greater than that of leukocytes and erythrocytes [15,16]. Approaches such as Vortex Chip leveraged this difference to preferentially trap CTCs [17]. While such size-based approaches are appealing at first, heterogeneity in CTC size [18,19,20] may cause these strategies to fail at capturing the full heterogeneity of CTCs. Enrichment strategies that leverage differences in the dielectric properties of cell membranes between CTCs and PBMCs have their own limitations. These include that cell concentrations must be kept low to facilitate discrimination, decreasing speed of the enrichment, and that the dielectric properties of cells and their sensitivity to electric fields change over time, convoluting the enrichment process [21].

Label-dependent methods target cell-surface molecules and can be divided into positive or negative enrichment strategies. Label-dependent, positive enrichment involves antibodies coupled to magnetic nanoparticles that target cell-surface molecules on CTCs. A popular example is the FDA-approved CellSearch platform (VeriDex LLC) that uses anti-EpCAM immunomagnetic antibodies to positively select for CTCs. The limitations are that EpCAM selection is not appropriate for cancers of non-epithelial origin such as sarcoma, melanoma, and brain cancer [22]. Additionally, cells are permeabilized as part of the staining protocol, which restricts the number of possible downstream analyses [13]. An alternative approach is to use a cocktail of antibodies for positive enrichment; however, studies demonstrating this strategy have been restricted by low recovery efficiency and specificity [23]. Moreover, epitopes targeted for positive enrichment are occupied by the antibodies used for selection, limiting their use in downstream fluorescence staining.

**Figure 1:**
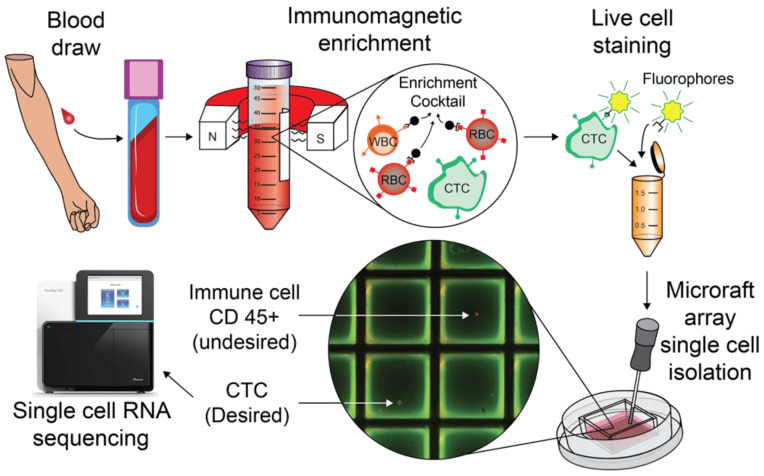
**CTC Isolation Workflow.** Up to 20 mL of melanoma patient blood is drawn. Using the StemCell Technologies EasySep Direct Human CTC Enrichment Kit, all undesired components of the blood are removed including red and white blood cells. Enriched CTCs are stained, live, via direct immunofluorescence, and then plated on a CytoSort Array. Cells that positively stain for the melanocyte cocktail and Hoechst, and negatively for CD45, are picked with a magnetic wand and deposited in a PCR tube with lysis buffer. Libraries are prepared for single-cell RNA sequencing according to the Smart-seq2 protocol and sequenced on a next-generation sequencing platform.

Label-dependent, negative enrichment for CTCs typically involves the removal of erythrocytes via Ficoll–Paque density gradient separation or RBC lysis, followed by an immunomagnetic depletion of CD45^+^ leukocytes, leaving behind enriched CTCs [24]. Targeting exclusively CD45 to negatively enrich CTCs has historically yielded a low purity population with high leukocyte contamination, as reviewed in Marsavela et al. 2018 [25]. In this study, we chose to utilize an enrichment cocktail in order to perform enrichment of CTCs that does not rely on physical properties or antigen expression of cancer cells (see Figure 1 for a schematic of the workflow of the study), which we believe is a more optimal approach for capturing a fuller measure of the heterogeneity of CTCs. Use of the enrichment cocktail allowed for the distinction of peripheral immune cells as well as any other autofluorescent material that may have survived the previous enrichments. Positively marked cells were retrieved via a novel approach using CytoSort single-cell arrays, which have never been applied towards CTC isolation. On average, the CTC isolation approach used in this study took about five hours with variation depending on the number of CTC’s identified during the picking process.

After successful enrichment of CTCs, there are many possible downstream analyses as has been reviewed elsewhere [26]. An important distinction is between bulk and single-cell methods. Cellular heterogeneity is a critical issue in cancer development and progression, and may explain treatment resistant cells and a lack of response to treatment [27]. Single-cell methods, as opposed to bulk methods, are critical to discovering and interrogating the heterogeneity of these cells. In the current study, we performed single-cell RNA sequencing (scRNA-seq) on our patient-derived CTCs to glean maximal information about cellular heterogeneity, including heterogeneity of gene expression (which is only available through RNA-based analysis). We selected Smart-seq2 as our scRNAseq library prep method [28]. Previous studies have shown Smart-seq2 provides the highest sensitivity to detect genes compared to other library prep methods including CEL-seq2, DROP-seq, MARS-seq, SCRB-seq, and SmartSeq1 [29]. Other approaches have focused on identification of DNA-mutational changes, which is beyond the scope of this work.

## 2. Materials and Methods

### 2.1. Patient Recruitment and Specimen Collection

Patients diagnosed with cutaneous melanoma, confirmed to begin treatment with immune checkpoint blockade immunotherapy, were enrolled at the Oregon Health and Science University Department of Dermatology Melanoma Multidisciplinary Clinic between 2 December 2019 and 2 February 2020. Trained phlebotomists drew up to 20 mL of blood in two heparinized vacutainers. Collections were taken at various time points throughout immunotherapy (Figure 4).

### 2.2. Ficoll-Paque Density Gradient Separation for Isolation of Healthy Control PBMCs

About ~10 mL of healthy control blood was collected in heparinized vacutainer tubes. Blood was diluted to 30 mL with phosphate-buffered saline (PBS) and slowly layered on top of 10 mL of Ficoll–Paque (1.084 ρ) (GE Healthcare, Chicago, IL, USA), and then spun at 800 g for 20 min (no brake) at 4 °C. The plasma fraction was collected, and then the buffy coat layer was collected and resuspended in 40 mL of wash buffer (RPMI 1640, 25 mM HEPES, and 0.5% FBS) and spun at 800 g for 10 min (low brake) at room temperature. The wash buffer was aspirated off, and the PBMC pellet was resuspended in freezing media (90% FBS, 10% DMSO). Cells were viably frozen using a “Mr. Frosty” to regulate the temperature decrease.

### 2.3. CTC Enrichment with StemCell Technologies EasySep Direct Human CTC Enrichment Kit

We utilized the StemCell Technologies EasySep Direct Human CTC Enrichment Kit (Catalog #19657) to enrich CTCs by depleting leukocytes and erythrocytes. All steps were performed in a sterile biosafety cabinet. We made various adjustments to the manufacturer’s protocol to attain a highly pure enriched population. Specifically, up to 20 mL of blood was added to a 50 mL conical. Then, 50 μL of enrichment cocktail (provided in kit) was added per 1 mL of blood. Blood plus enrichment cocktail was mixed slowly by inversion 10 times, then incubated for 5 min at room temperature. RapidSpheres were vortexed for 30 s before adding 50 μL of RapidSpheres per mL of blood and then mixed by inversion 10 times. EasySep Buffer (Ca- and Mg-free PBS with 2% FBS and 1mM EDTA) was added to double the volume and then mixed by inversion 10 times. The sample was placed in the “Easy 50” magnet, ensuring there was a clear view in the window. The sample was incubated at RT for 15 min. The enriched cell suspension was pipetted into a new 50 mL conical tube, being careful not to collect RBCs. Again, RapidSpheres were added at 50 μL/mL of sample, and the sample was mixed by inversion 10 times. The tube was again placed in the Easy 50 magnet and incubated at RT for 15 min. The entire clear fraction/enriched cell suspension was pipetted into a new 50 mL conical tube. The sample was centrifuged at 800 g for 15 min to pellet cells. The supernatant was aspirated, and the pellet was resuspended in 3 mL of EasySep Buffer and then transferred to a 5 mL flow tube. The flow tube was placed in the EasySep purple magnet and then incubated at RT for 10 min. The enriched cell suspension was poured into a 15 mL conical tube then centrifuged at 800 g for 5 min to pellet cells. The supernatant was again aspirated, then resuspended in 100 μL of freshly prepared blocking solution (3% milk in PBS, 0.5% BSA, and 0.02% NaN_3_).

### 2.4. Live Cell Staining

To differentiate CD45^+^ cells and CTCs in our sample, we performed a live cell stain using direct immunofluorescence (DIF) with an antibody cocktail (Supplementary Table S1). The cocktail included 1 uL of PRAME [30], MCAM [31], MCSP [32], c-Kit [33,34], PD-L1 [4,5], MART-1 [35], and CD45, and 0.5 uL of hoechst nuclear stain (20 mM). FC block (BD Bioscience, Franklin Lakes, NJ, USA) was added to the cells at a concentration of 1 µL FC block per 100 µL of blocking solution, and incubated for 15 min at 4 °C. Next, 1 µL of each antibody from Supplementary Table S1 and Hoechst stain (1 ug/mL) were added. Cells were incubated at 4 °C for 20 min in the dark. The cells were washed with 1 mL of staining buffer (0.5% BSA, 0.02% NaN_3_ in PBS) and spun at 300 g for 5 min. The cells were washed again with 1 mL of staining buffer and then centrifuged at 300 g for 5 min. Staining buffer was gently aspirated off, and the cells were resuspended in 200 µL PBS and plated on the CytoSort array, prepped with Corning Cell-Tak (final array volume 2.2 mL).

### 2.5. Prepping Cell Microsystems CytoSort Arrays with Corning Cell-Tak Cell and Tissue Adhesive

The sterile CytoSort Array was opened in a biosafety cabinet. Next, 2 mL of PBS was added and incubated at RT for three minutes and then aspirated out. This step was repeated and then 1 mL of 0.1 M Sodium Bicarbonate at pH 8.0 was mixed with 7.23 µL of Corning Cell-Tak, vortexed to mix, and applied to the CytoSort Array. This mixture was incubated for 20 min at RT and then aspirated off. The array was washed twice more with 2 mL of PBS, incubating for three minutes each, and then left in 2 mL of PBS until 200 µL of the enriched CTCs were plated. When plating, the enriched cell suspension was pipetted in a figure-eight pattern to more effectively distribute cells across the array, avoiding doublets.

### 2.6. CytoSort Array Picking

Following DIF live-cell staining, cell visualization and single-cell isolation were performed using the Cell Microsystems Motorized Release Device for inverted microscopes and single reservoir CytoSort arrays. The Motorized Release Device rests over a 4X, 5X, or 10X objective on an inverted microscope. At the push of a button, a needle at the top of the device moves upwards, ejecting one of the CellRafts from the array, sitting on the stage above. The array is composed of 12,000 individual 200 μm × 200 μm releasable CellRafts that can be visualized on an inverted microscope. These rafts have embedded magnetic nanoparticles, which are used to physically manipulate and deposit rafts containing adhered single cells into PCR tubes using a magnetic wand. We used an EVOS M5000 imaging system (cat. no. AMF5000) to visualize our cells under the 10X objective with bright field and three-color fluorescence imaging (DAPI, GFP, and Cy5 led light cubes). We defined CTCs as FITC/melanocyte cocktail positive, nucleated (Hoechst^+^), CD45^−^ cells. Single CTCs in CellRafts were ejected from the array using the device and deposited into PCR tubes containing 4 μL of Smart-seq2 lysis buffer to begin the process of scRNAseq library preparation.

### 2.7. Single-Cell Library Preps

Single-cell RNA-seq libraries were prepared following the Smart-seq2 protocol from Picelli et al. 2014. Some subtle differences in library prep thermocycler conditions were required to optimize the preps for patient-derived CTCs when compared to A375s or the “mini-bulk” positive control reactions, as per Smart-seq2’s recommendations. For single A375s, patient-derived CTCs, and PBMCs, samples were preamplified (Smart-seq2 step 14) for 18 cycles, corresponding to the expected 10 pg of input RNA from the initial cell lysis. “Mini-bulk” positive control preps were preamplified for 16 cycles, corresponding to the 100 pg of input RNA. After bead purification, library concentration was determined with a Qubit dsDNA HS Assay Kit (Thermofisher cat. no. Q32851, Waltham, MA, USA) and an Agilent Bioanalyzer High Sensitivity DNA kit (cat. no. 5067-4626, Santa Clara, CA, USA). Next, 1 ng of bead-purified, preamplified dsDNA library was loaded into the tagmentation reaction at step 28 of the Smart-seq2 protocol. The final amplification reaction at step 33 was carried out for 12 cycles. All steps not discussed here were carried out as described in Picelli et al. 2014.

### 2.8. A375 Single Cell Preps

A375 human melanoma cells were purchased directly from ATCC. Further, 1000 A375 cells from the same passage as the mini-bulk preps were stained using our live-cell staining cocktail and plated on a microarray prepped with Cell-Tak. Eight single, melanocyte cocktail+/Hoechst+ A375s were picked and deposited into Smart-seq2 lysis buffer, and single-cell libraries were prepared as recommended.

To isolate PBMCs for scRNAseq, 10 mL of healthy control blood was drawn, and separated using a Ficoll–Paque density gradient to enrich PBMCs, which were then viably frozen. Viably frozen cells were thawed, washed in media, and then live-cell stained using our cocktail and quantified. In total, 1000 stained PBMCs were plated on a microarray prepped with Cell-Tak. Then, 13 single, CD45+/Hoechst+ PBMCs were picked, and single-cell libraries were prepared following the Smart-seq2 protocol.

Single-cell library preparation was initiated within three months of specimen collection and cell lysis. Patient-derived CTCs have been reported to yield low amounts of RNA [36], making for a challenging single-cell library prep. Therefore, we analyzed libraries using the Qubit dsDNA HS Assay Kit (Thermofisher cat. no. Q32851) and an Agilent Bioanalyzer High Sensitivity DNA kit (cat. no. 5067-4626) on the library preps after Smart-seq2 steps 27 and 36 to decide which libraries had sufficient quality material to proceed. Libraries that contained a majority primer dimer product, indicated by a sharp peak between 75 and 150 bp on bioanalyzer trace were discarded. Successfully composed libraries were identified by a broad, tall peak between 200 and ~8000 bp as shown in Supplementary Figure S1.

### 2.9. A375 Mini-Bulk Preparations

To prepare the mini-bulk preps, total RNA was extracted from 1 million A375s using QIAGEN (Hilden, Germany) RNeasy mini kits (cat. no. 74004). Isolated RNA was quantified using the Invitrogen Qubit RNA HS Assay Kit (Thermofisher cat. No. Q32852), and quality control was performed on an Agilent Bioanalyzer using the Agilent Bioanalyzer RNA 6000 Pico Kit (Agilent cat. no. 5067-1513) to ensure the integrity of the RNA.

### 2.10. Data Analysis

Data analysis was performed using an adapted Snakemake (version 4.3.0, Johannes Köster, North Rhine-Westphalia, German) pipeline found at https://github.com/m-chang3/CTC_Pipeline (accessed on 19th May 2021). The paired, gzipped raw reads were trimmed using TrimGalore (v. 0.6.2). Initial quality control metrics were calculated using the FASTQC package in addition to the program Fastqscreen (with the Bowtie2 aligner specified). Hisat2 (version 2.0.4) mapped the reads to the hg38/GRCh.38 genome. The Hisat2 options required longer anchor lengths for the de novo discovery of splice sites and ignoring reads that do not align with their paired-end mates (--dta, --no-mixed, --no-discordant). The program featureCounts (part of the subread Anaconda package, version 1.6.4) was used to count reads mapped to individual features. A custom R script filtered this count matrix by mitochondrial contamination and feature biotype. Seurat version 4.0.1 analyzed the filtered counts matrix using a custom R script (Supplementary Information). Cells with greater than 20% mitochondrial transcripts or less than 250 unique genes detected were eliminated for further analysis.

To evaluate cellular heterogeneity (Figure 6e), we performed a sparse partial least squares discriminant analysis (sPLS-DA) on patient-derived CTCs (Figure 6e). Control cells (single and bulk A375s, PBMCs) were subsetted out, and patient CTCs were re-scaled and clustered. These expression values were then input to the sPLS-DA analysis tool (splsda()) from the R package “mixOmics” version 3.14. The results of this analysis were plotted and Euclidian distance of cells from their own cluster centroid were calculated. Finally, a Tukey test was implemented using these distances to determine if significant differences in cellular heterogeneity exist between patients. See the Github link for the code.

**Software Versions**:

TrimGalore: 0.6.2
fastqscreen: 0.14.0
Seurat: 4.0.1
Hisat: 2.0.4
featureCounts=subread version 1.6.4

## 3. Results

### 3.1. Negative Depletion Cocktail Yields High CTC Purity and Efficiency

**Figure 2:**
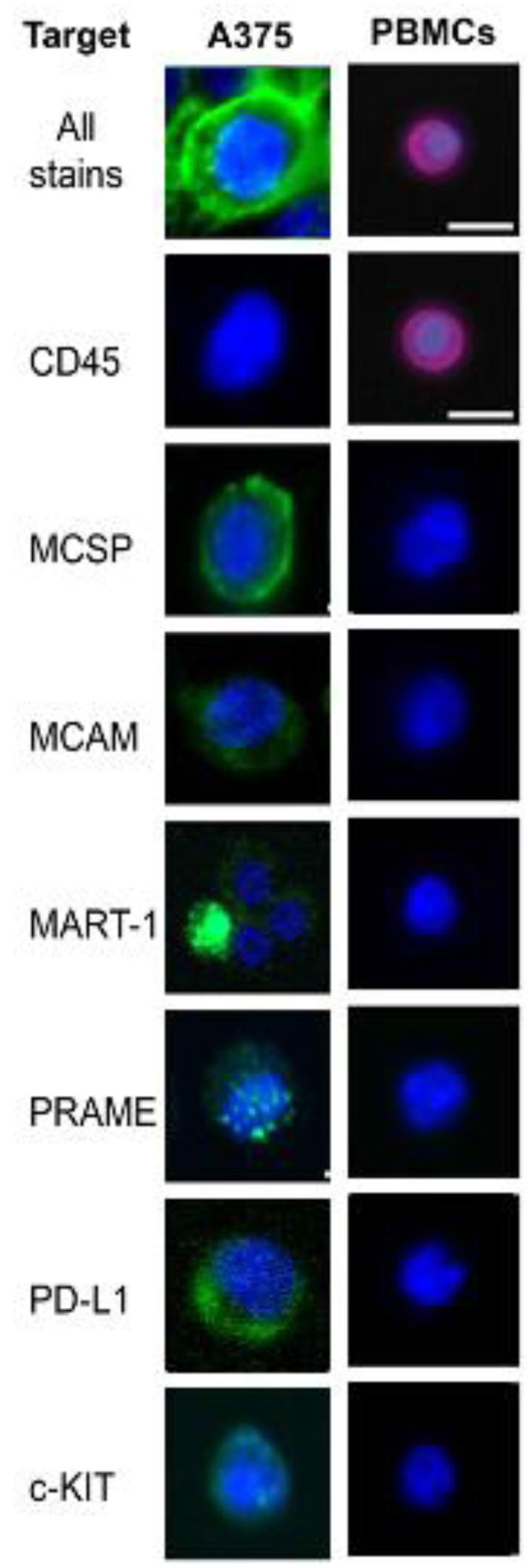
**Direct Immunofluorescence Live Cell Staining Cocktail Validation.** A375 and peripheral blood mononuclear cells (PBMCs) were stained with 1μL of each antibody to validate that the FITC-conjugated (green) melanocyte-specific markers stain melanoma cells but do not cross react with PBMCs, and ensure that APC-conjugated CD45 (red) does not cross react with melanoma cells. Some markers stained faintly and/or only on a subset of cells in the population, but were included in the final cocktail to enrich patient-derived CTCs with expression patterns that differ from these cell lines. Scale bars are 10 μm.

We first attempted to negatively enrich CTCs using a Ficoll–Paque (1.085 ρ) density gradient centrifugation to remove erythrocytes, followed by immunomagnetic depletion of leukocytes using anti-CD45 immunomagnetic particles (Invitrogen Dynabeads; Waltham, MA, USA) (Supplementary Information). We found, however, that there was high leukocyte contamination after the final enrichment, as has been previously discussed [26]. We then tested the StemCell EasySep Direct Human CTC Enrichment Kit, which utilizes a cocktail of anti-CD2, CD14, CD16, CD19, CD45, CD61, CD66b, and GlycophorinA magnetic antibodies to deplete erythrocytes and leukocytes simultaneously without the need for density gradient centrifugation or RBC lysis. To determine the recovery efficiency and specificity of our enrichment, we spiked 1000 A375 cells grown in tissue culture into 10 mL of healthy control blood and then performed the enrichment using the EasySep Direct Human CTC Enrichment Kit. The enriched population was stained with our live-cell staining cocktail (Figure 2) and plated on a CytoSort Array prepped with Corning Cell-Tak Cell and Tissue Adhesive.

**Figure 3:**
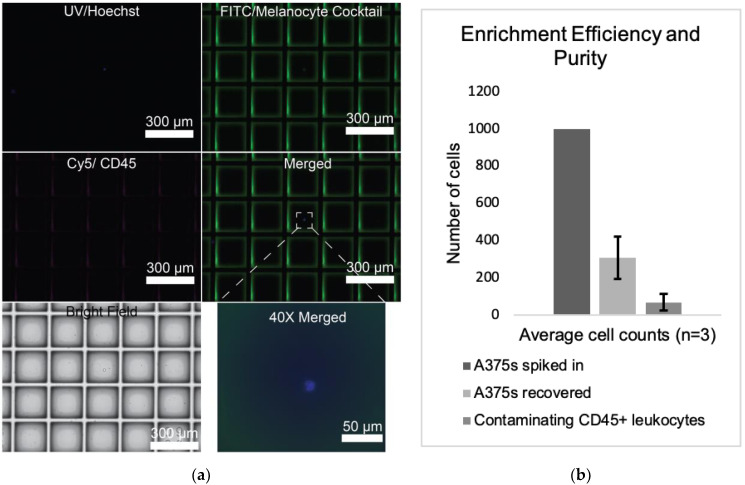
**Recovery Efficiency and Purity of Melanoma Cell Enrichment:** (**a**) The StemCell technologies EasySep Direct Human CTC Enrichment Kit combined with an antibody cocktail stain was used to identify and isolate melanoma CTCs on single-cell arrays. (**b**) A total of 1000 A375 cells grown in tissue culture were spiked into healthy control blood, enriched using the EasySep Direct Human CTC Enrichment Kit, stained using the live cell staining cocktail, plated on a CytoSort Array, and manually counted to determine the recovery efficiency (30.7%) and specificity (21.5% of nucleated cells on array are CD45^+^) of this method.

Cells were visualized and manually counted. This experiment was repeated three times, yielding an average recovery efficiency of 30.7%. In total, 110 out of 151 (72.8%) of the patient-derived CTC library preps that we attempted passed quality control metrics—a slight improvement over previously published observations (45–60%) [37,38]. The population was highly pure: 78.5% of nucleated cells stained positively for the melanocyte cocktail, while 21.5% stained positively for CD45 (Figure 3a,b). This enrichment process shows notable improvement over other CTC isolation methods such as the microfluidic based assay outlined by Lee et. al. [39]. While they report high purity and comparatively high enrichment efficiency of 61%, their approach is still below our captured efficiency rates and can only accept three milliliters of blood input per chip.

### 3.2. CTCs Are Present in Many Advanced Melanoma Patients

**Figure 4:**
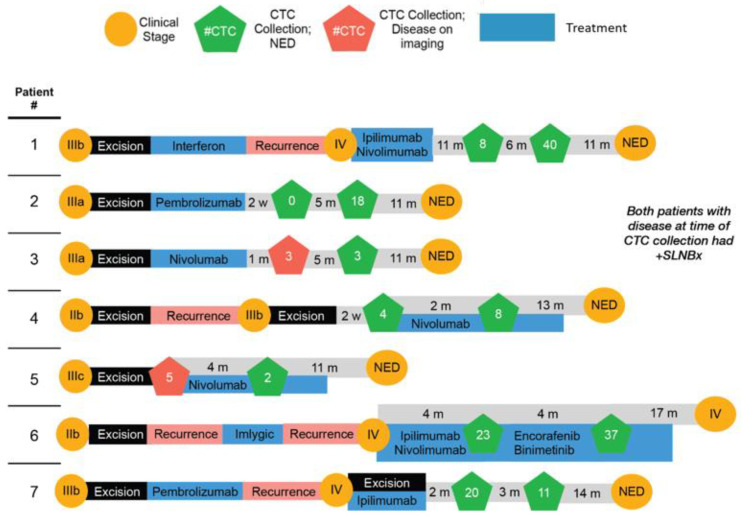
**Timelines of CTC Collection.** This illustration demonstrates collection times and the number of CTCs collected with accompanying clinical data including disease stage (yellow circles), surgical excisions (black rectangles), treatments (blue rectangles), and disease recurrence (pink rectangles.) Green pentagons indicate CTC collections taken at a time when patients had no clinical evidence of disease through diagnostic imaging. Red pentagons indicate CTC collections taken when patients had evidence of disease, such as a positive sentinel lymph node biopsy. NED; no evidence of disease. SLNBx; sentinel lymph node biopsy.

Following validation of the live cell-staining cocktail and enrichment protocol on melanoma cells grown in tissue culture, we tested the pipeline on the blood of melanoma patients that were undergoing immune checkpoint blockade (Figure 4). We successfully isolated 182 single melanoma CTCs from seven patients (20 mL of blood per collection) at various treatment time points (Supplementary Table S2) for a total of 12 collections. Individual cells were picked and deposited directly into 4 μL of Smart-seq2 lysis buffer (containing an RNAse inhibitor) and then flash frozen and stored in liquid nitrogen for subsequent library preparation.

### 3.3. A375 Validation of Smartseq-2 Single-Cell Analysis

We sought to contrast the transcriptional profiles of patient-derived melanoma CTCs against those of single A375 cells, “mini-bulk” positive controls, and healthy control PBMCs. We hypothesized this would help confirm their identity as cancer cells of melanocyte origin and elucidate the cellular heterogeneity of patient-derived CTCs. The mini-bulk positive controls (on 1 million A375 cells) and single A375 library preps were performed on cells prepared at the same passage. The important distinction between this mini-bulk library prep and single-cell preps is that the preamplification PCR was carried out for 18 cycles for all single-cell samples and 16 cycles for the mini-bulk prep. The decrease in cycles for the mini-bulk prep was proportional to the increase in input material, as recommended by the Smart-seq2 protocol [28]. Samples with the desired product were identified via based on the absence of small (<500 bp) fragments and a relatively sharp peak between ~1500 and 2000 bp on a bioanalyzer trace. Overall, 96 of the 98 library preps that passed the quality control metrics for PCR preamplification products previously mentioned also passed the final quality control analysis for the purified Tagmentation PCR product with a broad peak between 300 and 800 bp.With ~98% of libraries yielding high-quality reads, we are confident in the quality of sequencing data that can be pulled via this single-cell approach.

### 3.4. RNA Sequencing Helps Confirm Melanocytic Identity of Patient-Derived CTCs

**Figure 5:**
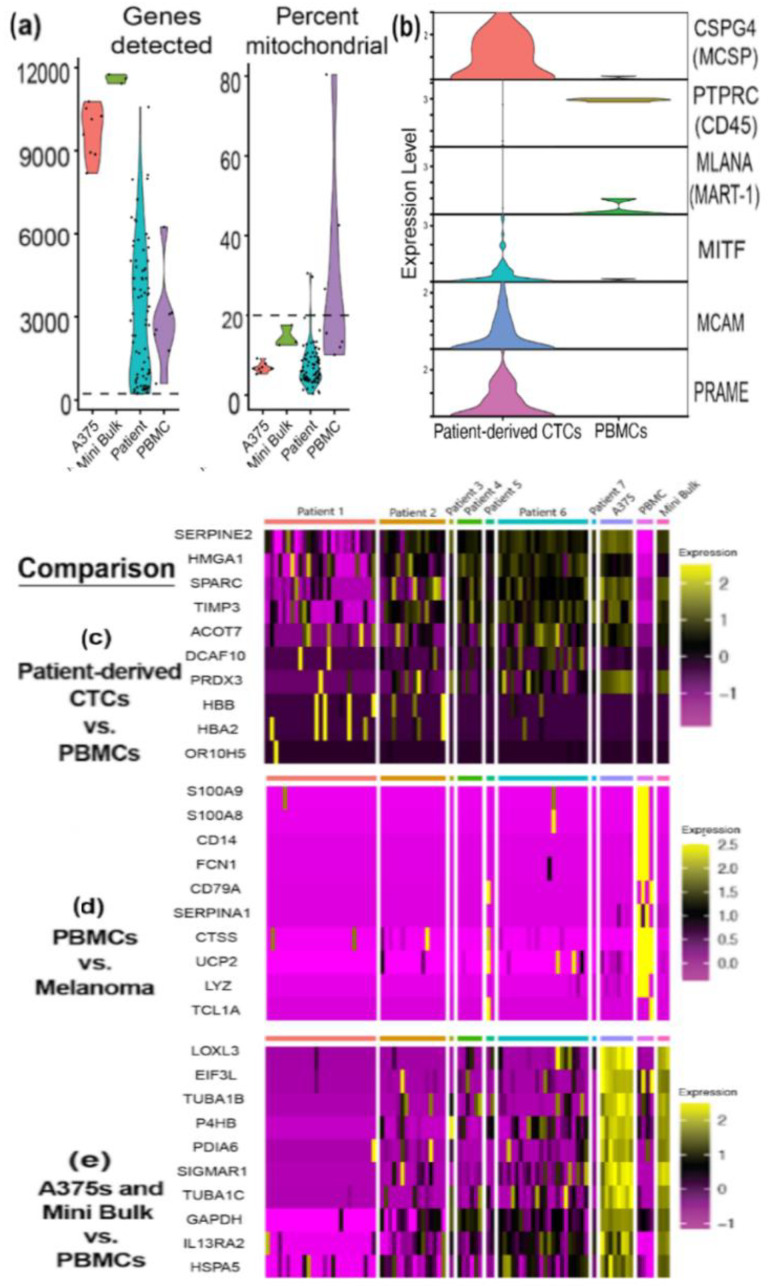
**Melanoma CTCs are enriched for melanocytic gene expression by next-generation sequencing analysis.** (**a**) The number of unique genes detected (left) and percentage of mitochondrial transcripts detected (right) for single A375 cells, mini-bulk preps, patient-derived CTCs, and PBMCs. Dashed lines indicate QC cutoffs used to remove low-quality cells. (**b**) Violin plot demonstrating expression levels of melanocyte-specific genes (*CSPG4* (MCSP), *MLANA* (MART-1), *MITF*, *MCAM*, *PRAME*), and *PTPRC* (CD45) in patient-derived CTCs compared to PBMCs. (**c**–**e**) Heat maps demonstrating the top 10 most differentially expressed genes when comparing (**b**) patient-derived CTCs to PBMCs, (**c**) PBMCs vs. all other cells (patient-derived CTCs, single A375s, mini bulk preps), and (**d**) library preps from cells grown in tissue culture (A375 single cells and mini bulk preps) vs. PBMCs. Expression levels are given as the log_2_ fold change.

We performed quality control of the next-generation sequencing (NGS) data by removing low-quality cells with greater than 20% mitochondrial transcripts or fewer than 250 unique genes detected. This left 75 (75/78, 96%) patient-derived CTCs, eight (8/8, 100%) single A375s, three mini-bulk preps, and four (4/7, 57%) PBMCs for analysis. The remaining cells showed an average of 3180, 9659, 11593, and 3756 unique genes detected for patient-derived CTCs, A375s, mini bulk preps, and PBMCs, respectively (Figure 5a). An average of 6.80, 6.87, 14.47, and 12.76%of the transcripts detected were of mitochondrial origin for patient-derived CTCs, A375s, mini bulk preps, and PBMCs, respectively (Figure 5a).

The top 10 differentially expressed genes in each cluster were used to generate heat maps. Comparing the top 10 differentially expressed genes between patient-derived CTCs and PBMCs yielded numerous hits with relevance to cancer development and progression (Figure 5b). Notably, SERPINE2 has been implicated in increasing the invasivity of slow-cycling melanoma cells [40]. *HMGA1* overexpression has been observed in a variety of tumors, and is associated with a poor prognosis [41]. SPARC is believed to promote melanoma cells to acquire mesenchymal traits—traits that can lead to increased metastatic dissemination [42,43]. TIMP3 acts as a tumor suppressor in melanoma, inhibits cell migration, and the expression has been shown to decrease as melanoma progresses [44]. Therefore, the cells contributing to the high expression of this gene may represent an early-stage cell population. *PRDX3* overexpression has been identified in various cancers, and is negatively correlated with survival in uveal melanoma [45]. Surprisingly, we observed increased expression of HBB and HBA2 in a subset of our patient-derived CTCs, possibly suggesting erythrocyte contamination; doublets in the CytoSort Array are more difficult to rule out when the contaminating cell is a red blood cell, as they are enucleated and do not stain with Hoechst.

As expected, numerous immune-related genes were detected in the top 10 differentially expressed genes of PBMCs when compared to the transcriptional profiles of all other (melanoma) cells (Figure 5c). The most differentially expressed, immune-related genes in PBMCs included *S100A8*, *S100A9*, *FCN1*, *CD14*, *CD79A*, *SERPINA1*, *CTSS*, *LYZ*, and *TCL1A*.

A375 single-cell and mini-bulk preps shared many differentially expressed genes when compared to PBMCs (Figure 5d). *LOXL3* and *IL13RA2* are among those with relevance to cancer. *LOXL3* overexpression in melanoma has been characterized, and some suggest its expression is a requirement for melanoma cell survival [46]. *IL13RA2* is another gene whose overexpression has been identified in melanoma and other cancer types, and it likely plays a pro-tumorigenic role in vivo [47,48]. The remaining top differentially expressed genes can broadly be categorized as those related to endoplasmic reticulum stress responses and protein folding.

To help confirm their identities as melanoma cells, we next compared the expression levels of common melanocyte-specific genes in patient-derived CTCs against the expression levels of these same genes in PBMCs. Violin plots illustrate that *CSPG4* (MCSP), *MITF*, *MCAM*, and *PRAME* were expressed robustly in patient-derived CTCs, but not PBMCs (Figure 5b). Conversely, *PTPRC* (CD45) expression was high in PBMCs, but low in patient-derived CTCs.

We performed statistical analyses (two-sided, unpaired t-tests for two-sample heteroscedastic datasets) on the expression levels of *PTPRC*, *PRAME*, *CSPG4*, *MCAM*, and *MITF* in patient-derived CTCs versus PBMCs. There was a statistically significant difference in expression of *PTPRC*, *CSPG4*, and *MCAM* between the two groups (*p*-values of 0.038, 0.000061, and 0.00000141, respectively). *PRAME* was nearly statistically significant with a *p*-value of 0.065. *MITF* expression was not significantly different between patient-derived CTCs and PBMCs. As a proof of principle, we performed the same statistical test comparing the expression level of various housekeeping genes (Supplementary Figure S2) in patient-derived CTCs vs. PBMCs and found no statistically significant difference in expression for *RPLP0*, *GAPDH*, and *HMBS* (*p*-values of 0.116, 0.124, and 0.247 respectively). One housekeeping gene, *ACTB*, did have a significant difference in expression between the two groups (*p*-value 0.041), though this gene has been shown to be upregulated in a variety of cancers, including melanoma [49]. In summary, the significant increase in expression of melanocyte and melanoma-related genes helps confirm the identity of our patient-derived CTCs as melanoma.

### 3.5. RNA Sequencing Analysis Reveals Expression of Melanoma-Related Genes in Melanoma CTCs and Heterogeneity of Gene Expression

**Figure 6:**
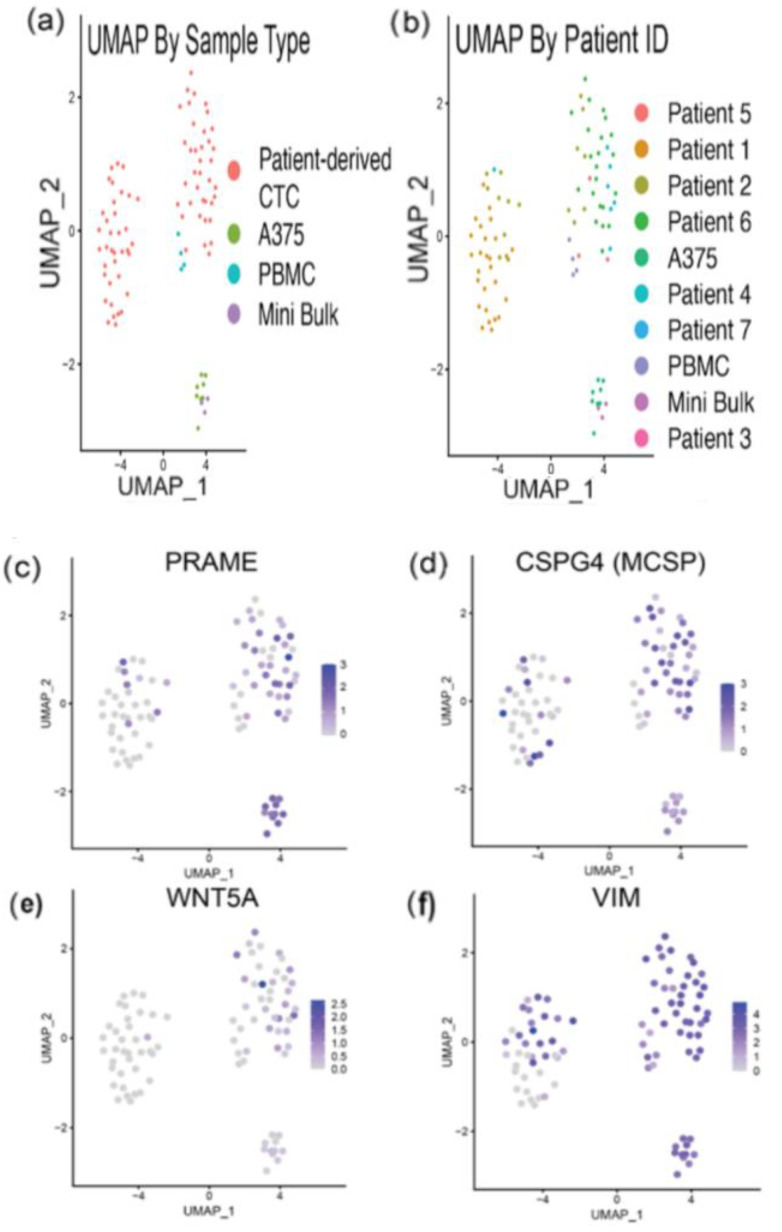
**NGS Data Analysis Demonstrates Substantial Cellular Heterogeneity:** (**a**) UMAP plot demonstrating clustering of cells colored by cell type. (**b**) UMAP plot demonstrating cell clustering colored by patient ID. (**c**–**f**) Feature plots of (**c**) *PRAME* and (**d**) *CSPG4* (MCSP) transcript expression demonstrate that some patient-derived CTCs express one, both, or neither of these targets, highlighting the importance of using multiple markers in a live-cell staining panel for CTC isolation. (**e**) Feature plot of WNT5a expression suggests aggressive, later-stage melanoma cells with a metastatic phenotype may exist among the population. (**f**) Feature plot of VIM expression, demonstrates some patient-derived CTCs may have undergone EMT and switched to an aggressive phenotype.

We wanted to investigate the expression levels of various melanoma-related genes across the cells in our dataset. We generated feature plots of the expression level of all targets in the live-cell staining panel (Supplementary Figure S3). UMAP dimensionality reduction demonstrated that the PBMCs clustered closely with other PBMCs, while single A375s and mini-bulk preps clustered together, as expected (Figure 6a). The patient-derived CTCs formed two major clusters, on the left and right of the UMAP plot. Patient-derived CTCs clustered less densely than the other cell types, suggesting substantial cell-cell heterogeneity. Notably, all cells from patient one clustered exclusively in the left group, while CTCs from patients two and four clustered within both patient-derived CTC clusters, suggestive of intra- and interpatient heterogeneity (Figure 6b). Looking at *PRAME* and *CSPG4* (MCSP) in particular, we detected transcripts of one, both, or neither of these two markers in our patient-derived CTCs (Figure 6c,d). Taken together, these findings highlight the importance of using a large cocktail of antibodies to stain for our CTCs, as expression of these targets is highly variable across samples.

We next investigated WNT/Beta Catenin signaling in our samples, as this pathway has been shown to affect melanoma cell proliferation and invasion, immunomodulation, and response to therapies [50]. WNT5a is involved in non-canonical WNT signaling, and can drive the switch to a metastatic phenotype and cytoskeleton remodeling in melanoma [51,52,53]. Notably, WNT5a expression has been shown to increase during melanoma progression, and correlates with poorer outcome [54]. Secreted WNT5a in the tumor microenvironment may also have an immunomodulatory role [55]. We observed heterogeneity in expression of *WNT5a* in our samples, which may represent a heterogeneous population of aggressive, later-stage melanoma cells that have switched to a metastatic phenotype (Figure 6e). Among our patient CTCs, we observed that CTC’s from patient 6 had notably higher WNT5a expression than the rest of the population (Supplementary Figure S4). Variable expression of WNT5a within patient 6’s CTCs also shows the intrapatient heterogeneity in gene expression within CTC populations.

Vimentin is an intermediate filament protein, normally expressed in mesenchymal cells. It is now recognized as a marker for cells that have undergone EMT [56]. Overexpression of vimentin has been demonstrated in metastatic melanoma, and correlates with a poor prognosis [57]. We then evaluated the distribution of *VIM* expression in our samples. We found high heterogeneity in *VIM* expression (Figure 6f). High *VIM* expression suggests that some of our patient-derived CTCs may have undergone EMT and switched to an aggressive phenotype.

**Figure 7:**
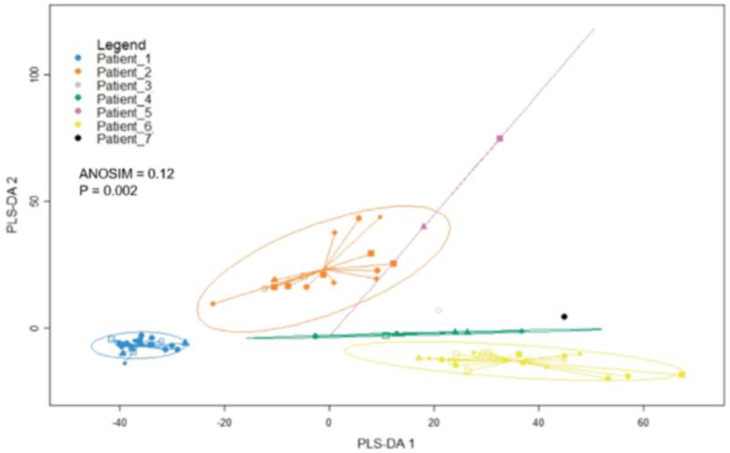
**Sparse Partial Least Squares Discriminant Analysis of Patient-Derived CTCs:** analysis of similarity (ANOSIM) demonstrates low interpatient heterogeneity (melanoma CTCs isolated from different patients are relatively similar to one another), but variable intrapatient heterogeneity.

One of the goals of this study was to develop a method for CTC isolation that captures maximal cellular heterogeneity. To test the heterogeneity of the collected CTCs, we performed a sparse partial least squares discrimination analysis (sPLS-DA) (Figure 7). Briefly, control cells (single and bulk A375s, PBMCs) were placed into subsets and patient CTCs were re-scaled and clustered. These expression values were then input to the sPLS-DA analysis tool from the R package “mixOmics,” version 3.14. The results were plotted, and the Euclidean distance of cells from their own cluster centroid were calculated. To evaluate heterogeneity of these samples, we performed an analysis of similarity (ANOSIM). The ANOSIM test was repeated 999 times, and the dissimilarity index was calculated to be 0.12 with a *p* value of 0.002 for our patient-derived CTCs. This indicated that these patient-derived CTCs are relatively similar, with approximately 12% of (dimensionally reduced) distance values being significantly different between groups.

We next wanted to test if there were differences in heterogeneity within CTCs derived from each patient (i.e., statistically significant differences in intrapatient heterogeneity). To accomplish this, we performed a Tukey test using the Euclidean distance of cells from their respective cluster centroids and found significant differences in cellular heterogeneity between patient samples. As an example, the CTCs isolated from patients 2 and 6 were significantly more heterogeneous than the cells isolated from patient 1 (*p* < 0.05). In summary, these analyses demonstrated that the population of patient-derived CTCs in this study were rather similar to one another (low interpatient heterogeneity), while the CTCs isolated from some patients were significantly more heterogeneous than those isolated from others (variable intrapatient heterogeneity).

### 3.6. Copy Number Variation Analysis

**Figure 8:**
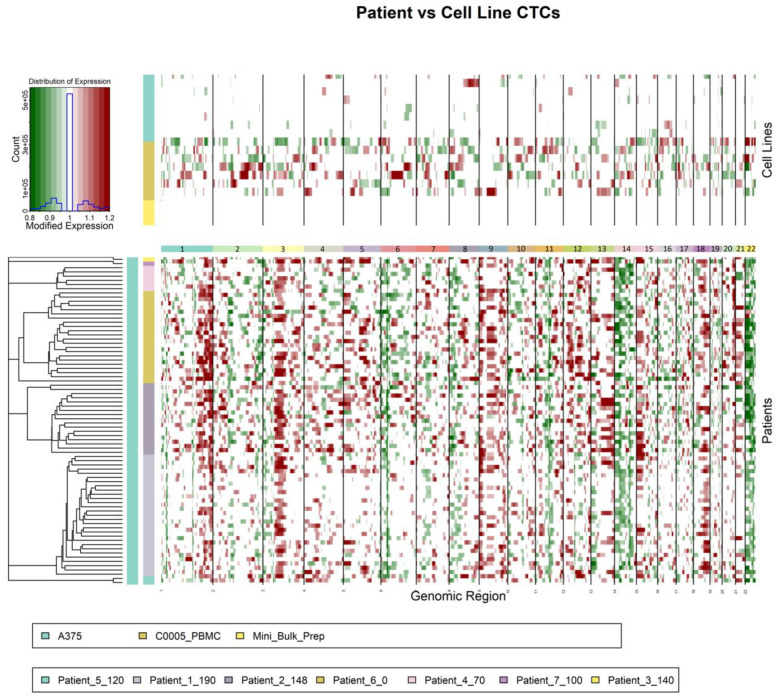
**Copy Number Variation Analysis Highlights Differences Between CTCs and PBMCs:** Chromosomal sequencing organized by chromosome number for patient CTCs (bottom), A375s (top), and PBMCs (top).

Copy number variation (CNV) CNV analysis of the PBMCs and CTC’s shows noticeably different chromosome compositions within chromosomes 1, 3, 6, 9, 11, 18, and 22 (Figure 8). This further validates the distinction between these two cell groups highlighted previously with genetic clustering. Interpatient comparison with the CNV analysis also shows considerable variation with chromosomes 6, 7, 11, 17, and 19, which highlights the genetic heterogeneity of the CTC population. CNV analysis of CTCs could serve as a useful tool in genetic counseling and chromosomal study of cancer patients. A larger number of CTCs would be required to confidently identify consistent chromosomal abnormalities from a patient’s cancer.

## 4. Discussion

Here, we demonstrated the feasibility of rapidly isolating and performing scRNAseq on patient-derived melanoma circulating tumor cells, isolated using a novel and inexpensive pipeline, and we showed several markers that could serve as biomarkers of melanoma CTCs. One of the aforementioned genes detected and measured in our CTC population, WNT5a, has been recently identified to play an important role in driving dormant cells towards melanoma metastasis [58]. The WNT5a gene serves as an example of one of many possible genes that can be tracked within CTCs to potentially predict disease behavior; changes in its expression in CTCs may herald transitions in tumor behavior. The pipeline we outlined entails a negative selection to enrich for CTCs followed by live cell staining using a large cocktail of antibodies to discriminate between CTCs and contaminating leukocytes, then isolating single CTCs using the CytoSort Array, and prepping NGS libraries using the Smart-seq2 protocol. NGS data analysis revealed that these CTCs express numerous genes that are specific to melanocytes and melanoma, increasing our confidence that we are indeed interrogating the correct cell type of interest. We set out to develop a method for enriching CTCs that captures maximal cell-to-cell heterogeneity. Further NGS data analysis highlighted substantial intrapatient CTC heterogeneity, and illustrated the wide variety of expression of genes involved in EMT, immunosuppression, metastatic dissemination, and tumor aggressivity.

This method is affordable, efficient, and requires minimal specialized equipment. The low cost of this approach obviates one roadblock to adoption of the methodology by other investigators. The method is also adaptable. Via single-cell isolation and sequencing, various genetic biomarkers (like WNT5a) can be identified and tracked to aid in clinical treatment. To study another rare cell population, such as prostate cancer CTCs, an investigator would simply need to change the antibody staining cocktail to be specific to the cell types of interest. This would allow for disease-specific genetic markers to be identified and tracked similar to the genes we outlined for melanoma. One might also consider adding an anti-glycophorin A antibody to the live cell-staining cocktail to help identify contaminating RBCs and avoid picking those wells, thereby reducing contamination. Future directions for this technology include expanding its implementation to investigate other cancer types and rare cells of interest, across a wide variety of disease states. This single-cell isolation method could be paired with a wide variety of alternate downstream analyses, including but not limited to: single-cell CHIP, ATAC sequencing and exome sequencing, and culturing/outgrowing viable cells.

While the methods we describe here have proven to be a robust strategy for CTC isolation, there are some limitations. As the enrichment, staining, picking, and library preparation steps are performed manually, this approach is rather labor intensive. Automation may help reduce the experimental burden of this approach (and indeed, Cell Microsystems has released an automated cell picking platform, the CellRaft AIR system), though this may come at significantly increased cost. Here, we selected scRNAseq as our downstream analysis method; however, previous studies have indicated that transcript and protein expression have a limited correlation (~40%) [59,60]. Therefore, proteomic or multi-omic approaches may more accurately characterize CTC behavior. Unfortunately, we were unable to use our NGS data to confirm BRAF mutation status in our CTCs compared to available clinical data from standard-of-care testing for clinically actionable mutations. This may be due to the low abundance of BRAF transcripts. CTCs are an inherently challenging target for scRNAseq due to the low quality of RNA input. In agreement with past studies, a large number of isolated single cells failed to produce a quality library for sequencing. In addition, the sample size presented here is limited, as our focus was also on detailing the improved methodologies to enable single-cell RNA seq analysis, and thus the specific genes that we have identified here as melanoma biomarkers may ultimately not be as useful to predict disease behavior in larger subsets when further tested.

Despite these shortcomings, the approach we outlined creates a unique opportunity for CTCs to become clinically viable as disease-tracking biomarkers. In addition to increased efficiency over microfluidics devices like those outlined by Lee et. al, our approach also presents an accessibility advantage few other methods can. One approach, which uses photoacoustic flow cytometry to detect and isolate CTCs from blood, can do so at extremely high efficiency (96%) [61]. However, this approach requires a considerable amount of specialized equipment and technical expertise that could not be broadly applied to oncology clinics. With relatively low input cost, basic technical equipment, and a simple to understand protocol, our assay offers a strategic balance between high-quality CTC isolation and clinically viable methods for CTC isolation. With this balance, CTCs can finally become a cancer biomarker accessible enough to practically influence provider decision making.

## 5. Conclusions

The patients we successfully collected CTCs from and performed scRNAseq on had no clinical evidence of disease at the time of collection. This invokes the concept of minimal residual disease; melanoma CTCs can be found at all disease stages and can persist long after treatment (as reviewed in Rapanotti et al., 2017 [32]). Previous work has shown that CTC counts are reduced after treatment, suggesting that this platform is sensitive enough to capture CTCs even in the context of low disease burden [62].

Following further validation, we believe the technologies and methods described here may be useful in a variety of clinical settings. First, we believe this technology holds the potential to elucidate clinically actionable mutations via a minimally invasive liquid biopsy. This may be especially useful in the case of patients who harbor non-surgically resectable tumors, or have micrometastatic disease, where the mutation status may otherwise remain unknown. Secondly, if CTCs are repeatedly collected throughout treatment, CTC enumeration and scRNAseq may reveal response or non-response to therapy. Lastly, such an approach may be paired with other forms of liquid biopsy, such as interrogating cell-free or circulating tumor DNA to build a more complete picture of a patient’s disease status. In conclusion, we believe this approach is a step forward along the path towards realizing the potential of liquid biopsies and precision medicine and can envision its utility in future clinical settings.

## Data Availability

The data presented in this study are available in this article.

## Supplementary Materials

The following supporting information can be downloaded at: https://www.mdpi.com/article/10.3390/cancers14194921/s1, Supplementary Methods. NGS data analysis scripts. Supplementary Figure S1: Representative Bioanalyzer 2100 Data From High Sensitivity DNA Chip Analysis of Libraries Before and After Tagmentation and the Final Enrichment PCR (QC performed at Steps 27 and 36 of Smart-seq2 protocol, respectively. Supplemental Figure S2: Expression of housekeeping genes across patient CTCs and controls. Supplementary Figure S3: Feature Plots of transcript expression of all Targets in CTC Live-Cell Staining Cocktail. Supplementary Figure S4: WNT5A Expression within patient CTC population. Supplementary Table S1: Direct Immunofluorescence Live Cell Staining Cocktail. Supplementary Table S2: Patient demographics.
