# Supplementary material for: Single-Cell Identification of Melanoma Biomarkers in Circulating Tumor Cells
Source: Cancers (Basel). 2022 Oct 8;14(19):4921. doi: 10.3390/cancers14194921 (PMC9564060; doi:10.3390/cancers14194921)
Supplement: Supplementary file 1 [file cancers-14-04921-s001.zip › cancers-1894632-supplementary.pdf]

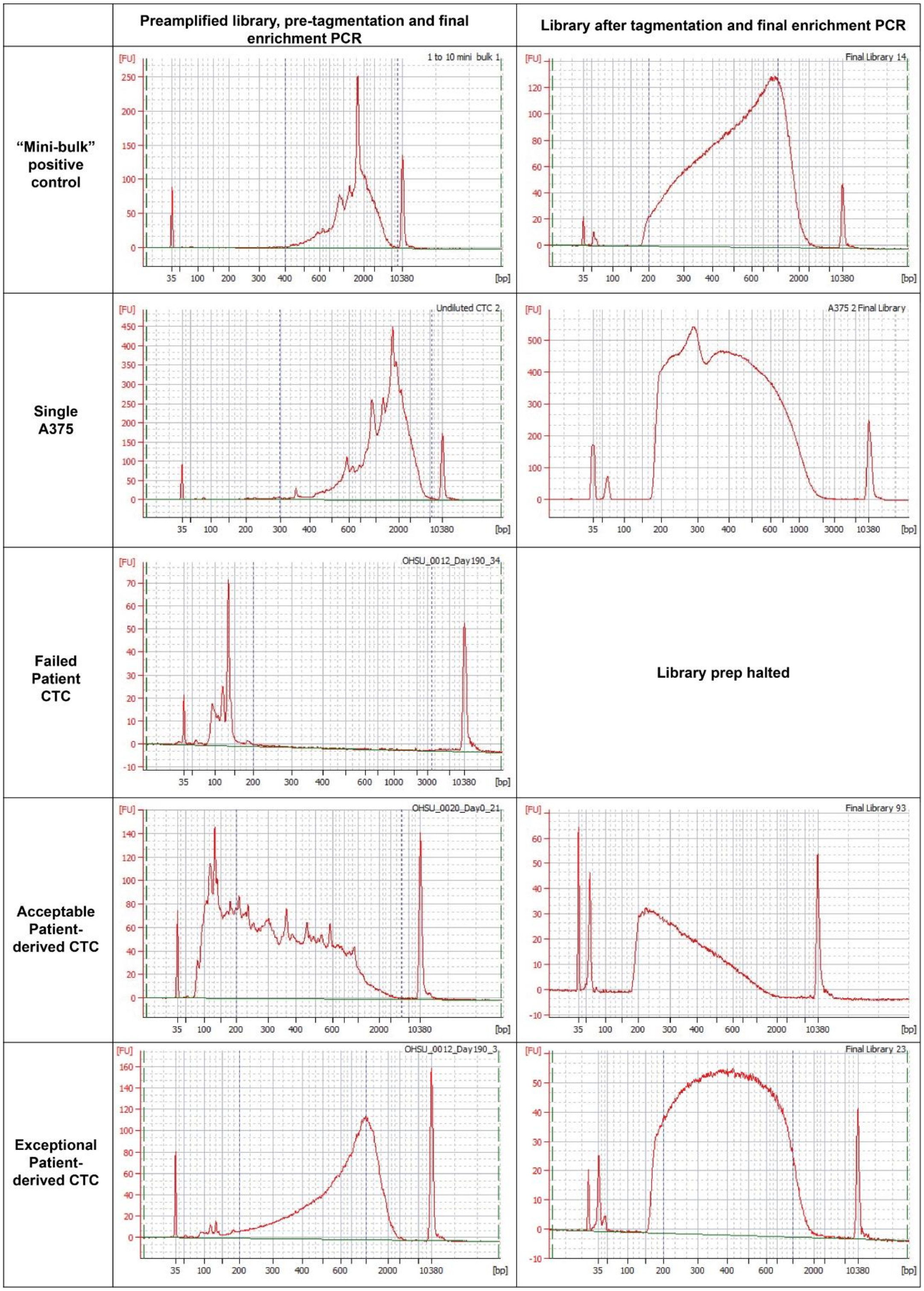

Supplementary Figure S1: Representative Bioanalyzer 2100 Data From High Sensitivity DNA Chip Analysis of Libraries Before and After Tagmentation and the Final Enrichment PCR (QC performed at Steps 27 and 36 of Smart-seq2 protocol, respectively): Each sample ("mini-bulk," single A375, and patient-derived CTC) was successfully sheared, then amplified into a sequencer-friendly library after tagmentation and the final enrichment PCR.

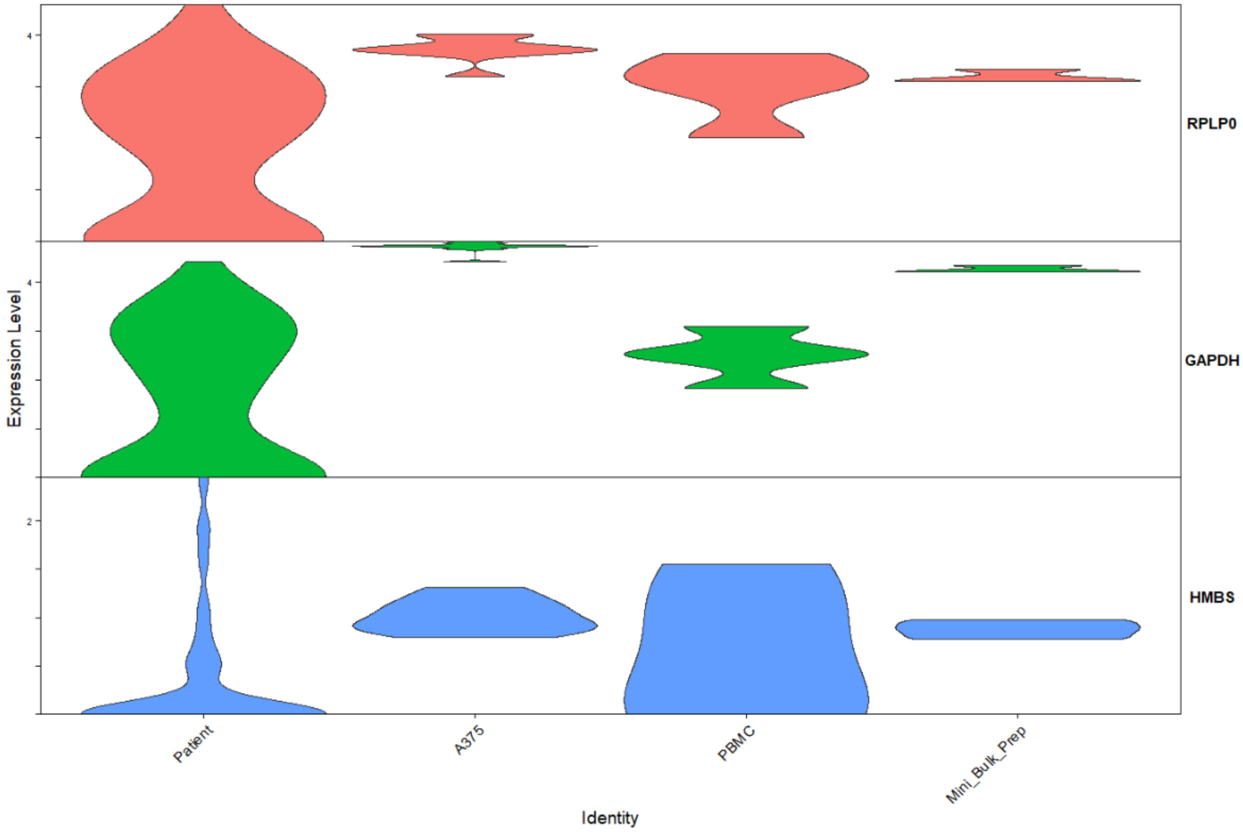

Supplemental Figure S2: Expression of housekeeping genes across patient CTCs and controls. Violin Plot of housekeeping genes (RPLP0, GAPDH, HMBS) showing inter-sample heterogeneity to demonstrate that RNA expression levels are affected evenly across patient samples and controls.

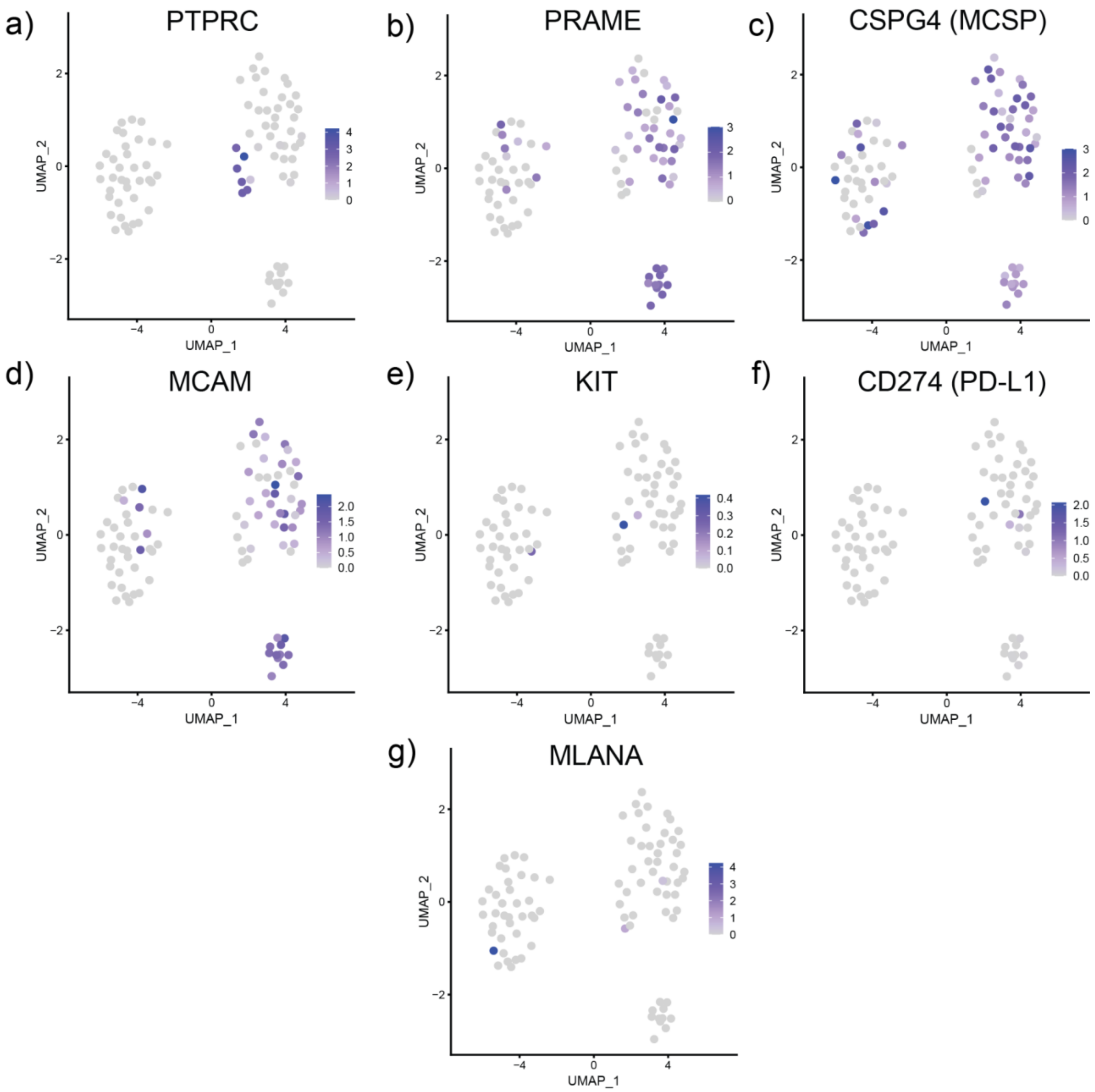

Supplementary Figure S3: Feature Plots of transcript expression of all Targets in CTC Live-Cell Staining Cocktail: *PTPRC* (CD45), *PRAME*, *CSPG4* (MCSP), *MCAM*, *KIT*, *CD274* (PD-L1), and *MLANA* transcript expression levels were evaluated across all samples in this study. **a)** As expected, PTPRC (CD45) expression was high in PBMC samples, and low in melanoma samples. The melanocyte and melanoma-specific targets demonstrated high variability in expression across samples, highlighting the importance of using a large cocktail of antibodies to stain CTCs during an enrichment.

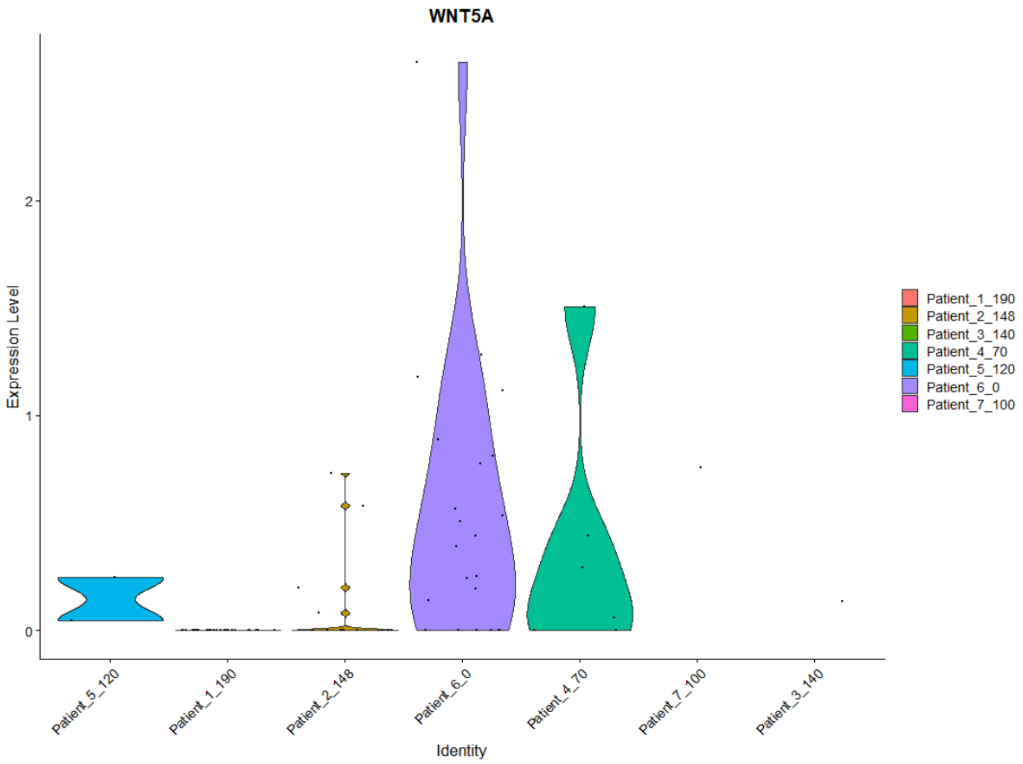

Supplementary Figure S4: WNT5A Expression within patient CTC population

Cell surface marker	Volume	Concentration	Vendor	Clone	Host/ Isotype	References	Catalog No.	Conjugate; Excitation max/emission max
**CD45**	1	1 uL/test	BD Biosciences	HI30	Mouse IgG1, κ	(Freeman et al. 2012) - not expressed on melanocytes	555485	APC; 650/660
**MCSP**	1	1 uL/test	BD Biosciences	9.2.27	Mouse BALB/c IgG2a	(Freeman et al. 2012; Kitago et al. 2009; Yang et al. 2004; Gray et al. 2015)	562413	Alexa Fluor® 488; 495/519
KIT (**CD117**)	1	1 uL/test	BD Biosciences	104D2	Mouse BALB/c IgG1	(Pilloni et al. 2011)	565172	BB515; 490/515
MART-1 (**MLANA**)	1	0.1 mg/mL	Biotium	A103	Mouse IgG1, κ	(Prieto and Shea 2011)	BNC880668	CF®488A; 490/515
PD-L1 (**CD274**)	1	1 uL/test	BD Biosciences	MIH1	Mouse BALB/c IgG1, κ	(Po et al. 2019)	558065	FITC; 494/520

MCAM (**CD146**)	1	1 uL/test	BD Biosciences	P1H12	Mouse IgG1, κ	(Freeman et al. 2012; Gray et al. 2015)	560846	FITC; 494/520
**PRAME** (PReferentially expressed Antigen in MElanoma)	1	50 ug/uL	Cell Signaling Technologies	E7I1B	Rabbit IgG	(Lezcano et al. 2018)	39509S	Alexa Fluor® 488; 495/519

Supplementary Table S1: Direct Immunofluorescence Live Cell Staining Cocktail. Six melanocyte-specific markers and one leukocyte marker were used to distinguish circulating tumor cells from contaminating leukocytes. Hoechst stain was added to visualize nuclei. 1 µl of each antibody was used for staining.

Patient #	Age	Sex	Tumor Site	Depth	Mutations	T	N	M	Stage at Dx	Concurrent Active Cancers
1	49	M	R flank	3.4	BRAFV600E	3b	1a	0	IIIB	NA
2	30	M	left lower back	1.8	BRAFV600E	2a	1a	0	IIIA	NA
3	54	M	R distal ulnar forearm	1.2	BRAFV600E	2a	1a	0	IIIA	NA
4	62	M	R chest wall	2.8	WT	3b	0	0	IIB	NA

5	44	F	Right leg x 2	3.3	BRAFV600E	3b	1a	0	IIIc	NA
6	70	M	R popliteal fossa	3.1	BRAFV600E, CHEK2 c.470C	3b	0	0	IIB	Lymphoma, clear cell renal carcinoma
7	68	F	R earlobe	1.15	HRASG13E (subclonal)	2a	1c	0	IIIB	NA

Supplementary Table S2: Patient demographics

NGS data analysis scripts are available via Github: https://github.com/m-chang3/CTC_Pipeline
